# Diabetes and Cancer‐Specific Survival: A Nationwide Population‐Based Cohort Study Across Multiple Cancer Sites

**DOI:** 10.1002/cnr2.70615

**Published:** 2026-06-18

**Authors:** Petrus Lohi, Jari Haukka, Leo Niskanen, Anssi Auvinen

**Affiliations:** ^1^ Tampere University, Faculty of Social Sciences Tampere Finland; ^2^ Department of Public Health University of Helsinki Helsinki Finland; ^3^ Department of Endocrinology, Päijät‐Häme Central Hospital Lahti Finland; ^4^ University of Eastern Finland Kuopio Finland

**Keywords:** cancer, cohort, diabetes mellitus, survival

## Abstract

**Background:**

Cancer patients with concurrent diabetes generally show poorer survival compared with nondiabetic patients.

**Aims:**

We examined the relationship between Type 2 diabetes and cancer‐specific survival in the 13 most common cancer sites within a large population‐based cohort in Finland. We hypothesized that survival would be lower among patients with diabetes across cancer types.

**Methods and Results:**

The cohort, derived from the Finnish CARING Project, included 49 799 incident cancer cases: 25 899 in patients with diabetes and 23 900 in nondiabetic patients. The mean age at diagnosis was 72.2 years, and 40.9% were women. During a mean follow‐up of 3.8 years (188 329 person‐years), 13 602 cancer deaths occurred. The impact of diabetes on prognosis varied by cancer type. Survival was substantially lower for stomach, endometrial, and lympho‐hematological cancers, and slightly lower for colon and breast cancer. Conversely, patients with diabetes had higher survival for liver and rectal cancer. No notable differences were observed for pancreatic, bladder, kidney, prostate, lung, or nonmelanoma skin cancer.

**Conclusion:**

Future studies should focus on the biological and metabolic factors influencing cancer survival in patients with diabetes and on how treatment strategies can be optimized to reduce excess cancer mortality in this patient group.

**Abbreviations**
IGF: insulin‐like growth factor
OAD: oral antidiabetic
SII: Social Insurance Institution

## 1 Introduction

Obesity and Type 2 diabetes are known drivers of cancer progression [1]. According to a Danish study, 35% of people will develop diabetes during their lifetime, 44% will be diagnosed with cancer, and 15% will develop both [2]. In the UK, cancer has already surpassed cardiovascular disease as the leading cause of death among people with diabetes [3].

The association between Type 2 diabetes and cancer has been widely researched, and ample evidence has shown a link between the two diseases [4, 5, 6]. Metabolic changes in diabetes are postulated to enhance tumor proliferation. This is likely mediated mainly by hyperglycemia and hyperinsulinemia, but other hormones, growth factors, and free fatty acids may also play a role. Dysregulation of the insulin‐like growth factor (IGF) system, as well as various signaling pathways activated by inflammatory cytokines, adipocytokines, and other adhesion molecules, have been suggested to mediate the link between diabetes and cancer [7]. Multiple signaling pathways are involved in cancer development and in the process of acquiring the hallmarks of cancer that include abnormal survival, proliferation, metabolism, and angiogenesis [8]. The PI3K/AKT/mTOR pathway plays a central role in cell cycle and survival, apoptosis, and glucose metabolism [9]. In malignant pathologies, the dysregulation and hyperactivation of the mTOR cascade frequently drive unchecked cellular proliferation and enhance tumor survival [8, 9]. Inhibition of the pathway affects also immune function with implications for tumor immunology [10].

Patients with diabetes also have higher all‐cause mortality, while the evidence regarding cancer‐specific mortality remains inconclusive and controversial [11, 12]. Correspondingly, cancer patients with preexisting diabetes appear to have lower survival compared to those without diabetes [13, 14, 15]. Several underlying causes for the lower cancer survival in patients with diabetes have been proposed, including increased tumor cell proliferation due to hyperglycemia and hyperinsulinemia, differences in treatment of cancer, diabetes‐related co‐morbidities, and their effect on treatment decision or response to cancer treatment [15, 16]. There is also evidence suggesting that poor glycemic control in diabetes is linked to poorer cancer outcomes [17].

There is limited evidence on all‐site cancer survival in patients with diabetes [13]. While cancer survival tends to be lower overall, it varies considerably by cancer type. Few studies have investigated cancer‐specific survival across multiple cancer types, and there are gaps in the literature regarding some of the most common cancers.

We investigated the relationship between Type 2 diabetes and cancer‐specific survival in a large population‐based cohort in Finland. The aim was to evaluate the relationship between diabetes and cancer‐specific survival across the 13 most common cancer sites. We hypothesized that cancer‐specific survival would be lower among patients with diabetes for the reasons mentioned earlier.

## 2 Materials and Methods

### 2.1 Study Population

The original study cohort was constructed based on the Finnish CARING Project [18] and the population of the current study comprised all incident cancer cases in the original study population (*N* = 49 799) analyzed earlier by Lohi et al. [19].

The original study population included 185 258 patients with diabetes mellitus, as well as a reference group matched 1:1 to the diabetes cohort based on sex, age (±1 year), and hospital district. The diabetes group was identified as individuals who had purchased and received reimbursement for at least one insulin prescription (ATC code A10A) [20] and/or an oral antidiabetic (OAD) prescription (ATC code A10B) between January 1, 1997, and December 31, 2010. Individuals in the reference group had no recorded insulin or OADs purchases. Prescription data, including the date of purchase, quantity, and ATC code, were obtained from the Social Insurance Institution (SII) (permission Kela 16/522/2012). The cohort comprised all insulin users in Finland during this period, along with a 50% random sample of OAD users, as restricted by the SII for administrative reasons.

Cancer data were obtained from the population‐based Finnish Cancer Registry, with 97% completeness of coverage for all solid cancers in Finland [21]. The data included information on patients' sex and age at diagnosis, as well as the cancer's primary site (ICD‐O‐3 code) and date of diagnosis. Information on place of residence (hospital district), socioeconomic status, and dates and causes of death were obtained from Statistics Finland (permission TK‐53‐214‐12). Socioeconomic status was reported in the following categories: self‐employed, upper‐level employees, lower‐level employees, manual workers, students, retirees, and others. All data from the registers were anonymous, the researchers had no information about their identities, and there was no contact with the study population. According to Finnish law, no consent from patients is required for purely register‐based studies.

### 2.2 Ascertainment of Outcomes and Covariates

Specific cancer types were defined based on the topography and morphology codes of the International Classification of Diseases for Oncology (ICD‐O‐3). The primary outcome of this study was death due to specific cancer types (C00‐C97), excluding non‐melanoma skin cancers.

We analyzed the cancer‐specific survival of the most common cancer types, including cancers of the stomach, colon, rectum, liver and intrahepatic bile ducts, pancreas, bronchus and lung, hematopoietic and reticuloendothelial systems, nonmelanoma skin, kidney, urinary bladder, breast, endometrium, and prostate.

### 2.3 Follow‐Up

Follow‐up started on the day of cancer diagnosis and ended on death or December 31, 2017, which was used as the censoring date. The underlying cause of death had to match the type of cancer diagnosed at the beginning of follow‐up.

### 2.4 Statistical Analysis

Survival after cancer diagnosis was modeled using Poisson regression. The outcome was death from a specific cancer type, and the length of follow‐up was used as an offset term. We obtained point estimates and 95% confidence intervals for ratio and difference effects by comparing patients with and without diabetes using g‐computation. The following variables were included in the models: age, sex, calendar year, time since cancer diagnosis, socioeconomic group, and hospital district. Each cancer type was analyzed individually. Calculations were performed using R‐language [22] and risk Communicator package [23].

## 3 Results

A total of 25 899 cancer cases occurred in the diabetes group and 23 900 in the nondiabetic group (Table 1). Among people with diabetes, 23 608 used only oral diabetes medications, 617 used both oral medications and insulin, and 1674 used insulin only. Overall, the average age at diagnosis was 72.2 years, and 40.9% of the study population were women. Of these cases, 8720 were other than the most common cancer types (see Materials and Methods) and were excluded from the analysis.

**TABLE 1:** Basic characteristics of study population.

	Diabetes	Control
Total, *n*	25 899	23 900
Female sex, *N* (%)	10 832 (41.8)	9555 (40.0)
Socioeconomic group (%)		
Upper‐level employees	1230 (4.7)	1583 (6.6)
Self‐employed	1302 (5.0)	1250 (5.2)
Lower‐level employees	2081 (8.0)	2238 (9.4)
Manual workers	2705 (10.4)	2563 (10.7)
Students	131 (0.5)	122 (0.5)
Retired	16 193 (62.5)	14 329 (60.0)
Others	2257 (8.7)	1815 (7.6)
Cancer type, ICD‐10 code (%)		
Stomach (C16)	720 (2.8)	581 (2.4)
Colon (C18)	1722 (6.6)	1446 (6.1)
Rectum (C20)	793 (3.1)	700 (2.9)
Liver (C22)	921 (3.6)	342 (1.4)
Pancreas (C25)	1896 (7.3)	826 (3.5)
Lung (C34)	2294 (8.9)	2155 (9.0)
Lympho‐hematological (C42)	1092 (4.2)	1164 (4.9)
Skin nonmelanoma (C44)	2163 (8.4)	2186 (9.1)
Breast (C50)	2640 (10.2)	2910 (12.2)
Endometrium (C54)	899 (3.5)	583 (2.4)
Prostate gland (C61)	4453 (17.2)	5495 (23.0)
Kidney (C64)	965 (3.7)	656 (2.7)
Bladder (C67)	799 (3.1)	678 (2.8)

**FIGURE 1:**
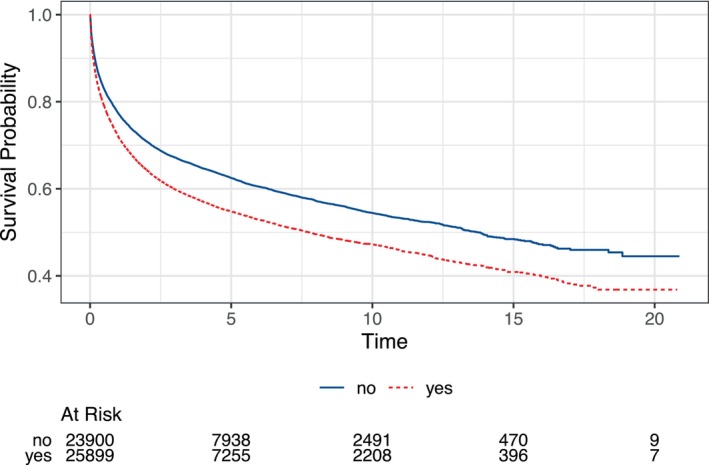
Kaplan–Meier curve for cancer‐specific survival for diabetes and nondiabetes groups.

In total, 13 602 cancer deaths were observed, and 188 329 person‐years were accumulated, with a mean length of follow‐up of 3.8 years. Of the cancer deaths, 13 142 were due to the cancer types we studied and were thus included in the analysis. The Kaplan–Meier curve demonstrated reduced cancer‐specific survival in the diabetes group compared with the reference group (Figure 1). Similar analyzes were conducted for each cancer type, and the corresponding Kaplan–Meier curves are provided in the Supporting Information (Figure S1).

**FIGURE 2:**
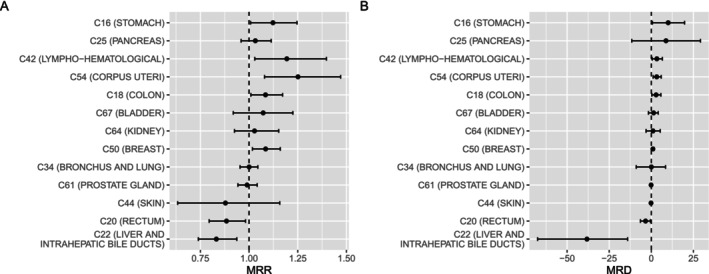
Mortality rate ratios (A) and mortality rate differences (B) for diabetes with 95% confidence interval, adjusted for age, sex, calendar year, socioeconomic group, and hospital district. Cancer types are categorized using ICD‐10 codes.

The adjusted mortality rate ratio (MRR) for patients with diabetes was substantially elevated (> 1.1) for cancers of the stomach, endometrium, and lympho‐hematological system (Table 2). The MRRs were also slightly elevated for colon and breast cancers. Whereas the MRR was increased for several cancer types, survival from liver and rectal cancer was higher in patients with diabetes compared to those without diabetes. No material differences were found in the survival of patients with pancreatic, bladder, kidney, prostate, lung, or nonmelanoma skin cancer (Figure 2).

**TABLE 2:** Number of cancer cases by cancer type, number of cancer‐specific deaths in diabetes group and comparison group, adjusted mortality rate ratios (MRR) and mortality rate differences (MRD) for each cancer type with 95% confidence intervals.

Cancer type	Number of cases		Number of deaths		MRR	MRD
	Diabetes	No diabetes	Diabetes	No diabetes		
Stomach (C16)	720	581	438	355	1.12 (1.01, 1.25)	10.03 (0.68, 19.81)
Colon (C18)	1722	1446	612	507	1.08 (1.01, 1.17)	2.86 (0.32, 5.71)
Rectum (C20)	793	700	247	217	0.88 (0.80, 0.98)	−3.38 (−6.42, −0.52)
Liver (C22)	921	342	664	275	0.83 (0.74, 0.94)	−38.21 (−67.55, −14.08)
Pancreas (C25)	1896	826	1698	721	1.03 (0.96, 1.11)	8.74 (−11.60, 29.26)
Lung (C34)	2294	2155	1783	1699	1.00 (0.95, 1.05)	0.06 (−8.92, 8.45)
Lympho‐hematological (C42)	1092	1164	405	395	1.19 (1.03, 1.40)	3.30 (0.54, 6.61)
Skin nonmelanoma (C44)	2163	2186	30	29	0.88 (0.63, 1.16)	−0.15 (−0.55, 0.17)
Breast (C50)	2640	2910	404	348	1.09 (1.02, 1.16)	0.96 (0.20, 1.76)
Endometrium (C54)	899	583	178	86	1.25 (1.08, 1.47)	3.22 (1.10, 5.88)
Prostate gland (C61)	4453	5495	541	632	0.99 (0.94, 1.04)	−0.12 (−0.66, 0.47)
Kidney (C64)	965	656	315	225	1.03 (0.93, 1.15)	1.03 (−3.09, 5.32)
Bladder (C67)	799	678	168	170	1.07 (0.92, 1.23)	1.39 (−1.65, 4.13)

*Note:* Adjusted for age, sex, calendar year, socioeconomic group, and hospital district. Cancer types are categorized using ICD‐10 codes.

We checked interactions between diabetes and sex, and diabetes and age, but did not detect any essential interactions in the sense of Breslow and Day (1980) [24].

## 4 Discussion

This study aimed to elucidate cancer‐specific survival among patients with Type 2 diabetes compared to matched nondiabetic individuals with cancer. We found that preexisting diabetes was generally associated with a decreased cancer‐specific survival. However, the impact of coexisting cancer and diabetes on patient prognosis was not consistent but varied significantly across cancer types. Specifically, the most pronounced decrease in survival was observed in endometrial and lympho‐hematological malignancies, while in cancers of the rectum and liver, the diabetes group had an increased cancer‐specific survival. These findings highlight that the prognostic burden of diabetes is highly site‐specific and suggest that metabolic factors influence cancer outcomes differently depending on the primary tumor site.

Impaired glycemic control is the key feature of Type 2 diabetes and is associated with alterations in growth factor profile with involvement of VEGF, EGF, IGF‐1, FGF‐21, and TGF‐β1 [25]. These abnormalities are important for the development of diabetic complications, but may also have a role in cancer development and progression [25, 26]. In the context of obesity and Type 2 diabetes, these growth factor signals are primarily mediated through the PI3K/AKT/mTOR pathway, which is altered across multiple tissues [27]. Specifically, in Type 2 diabetes, chronic hyperinsulinemia and nutrient excess lead to sustained mTOR activation, which intensifies insulin resistance but also creates a metabolic environment that promotes tumorgenesis [27, 28]. It affects the cell cycle and survival, apoptosis, and metabolism, that is, several key features in cancer development [9]. The alterations in the pathway occur in multiple tissues, which makes it relevant for the broad spectrum of malignancies affected in Type 2 diabetes [9, 27].

A Swedish register study by Liu et al. is a key point of comparison for our results [29]. It reported on 16 123 patients with cancer and Type 2 diabetes, with a mean follow‐up of 7 years but a total of only 60 492 person‐years. Our data on the other hand cover 49 799 cancer cases (25 899 with diabetes and 23 900 without diabetes) with an average follow‐up of 3.8 years and a total of 188 329 person‐years. In this Swedish study, cancer‐specific survival among patients with diabetes was significantly lower than that of other cancer patients, both for all cancers combined and for 16 individual cancer types out of the 26 examined. The largest differences were observed for endometrial, upper aerodigestive tract, thyroid gland, non‐Hodgkin lymphoma, and breast cancers. Liu et al. covered a larger number of cancer types than we did, and notably, for each cancer type examined, diabetes was associated either with worse prognosis or with no significant difference but never with improved prognosis. Our results were not as consistent: for two types of cancer, we found that diabetes was associated with a better prognosis, whereas for the other sites, it appeared to have an adverse or no impact on cancer prognosis. However, in the Swedish study, the diabetes group consisted of patients hospitalized with diabetes, likely representing more severe cases, thereby limiting the generalizability to the broader diabetes population. Moreover, the study was conducted between 1961 and 2008. One must bear in mind the evolution of the concept of diabetes and its treatment. The diagnostic criteria were altered by lowering the threshold of fasting glucose and accepting HbA1c levels ≥ 48 mmol/mol (6.5%) as diagnostic of diabetes [30]. Increased awareness of the impact of metabolic control on outcomes implied more widespread screening; therefore, more diabetic subjects were diagnosed in the earlier stages. Treatment goals have also become more intense over the past two decades. Furthermore, the very early use of metformin, endorsed by most guidelines for newly diagnosed Type 2 diabetes, has become the dominant drug in monotherapy or in combination with other agents [31]. Metformin has been the subject of intense study due to its possible anti‐cancer effects [32]. Thus, it is likely that patients with diabetes in our population had better overall and metabolic health than those in the study by Liu et al., and therefore survival from some malignancies was improved.

In our study, the largest decrease in survival was observed in endometrial cancer and lympho‐hematological malignancies. The findings concerning endometrial cancer are in line with previous literature [33]. Non‐Hodgkin lymphoma and chronic lymphocytic leukemia have also been previously associated with reduced survival in patients with diabetes [34, 35]. Since chronic lymphocytic leukemia is the most common type of leukemia and non‐Hodgkin lymphoma is the most common lymphoma, these two together account for the majority of lympho‐hematological malignancies, according to statistics from the US National Cancer Institute [36]. Few studies have examined the impact of diabetes on cancer‐specific survival in other lympho‐hematological malignancies.

In our study, survival from breast, stomach, and colon cancers was also moderately decreased among patients with diabetes. Our findings concerning breast cancer align with a large Mendelian randomization study showing lower breast cancer survival among patients with diabetes, although a smaller multiethnic cohort study found no such association [37, 38]. This discrepancy might be explained by different population characteristics. Specifically, the comprehensive analysis by Escala‐Garcia et al. was restricted to European‐descendent individuals, in contrast to the smaller US cohort, which featured a more diverse ethnic composition. It is therefore logical that our findings align with those of Escala‐Garcia et al., given that both studies involve more homogeneous populations of European ancestry within the context of European healthcare systems.

Contrary to our findings, improved survival from gastric cancer was reported among patients with diabetes in a Taiwanese study [39]. However, their study included only patients with gastric cancer who had undergone gastrectomy. Owing to the selected subgroup, their results are not directly comparable to our findings and may not be readily generalizable to all patients with gastric cancer.

In the literature on colorectal cancer, a meta‐analysis reported slightly decreased survival in patients with diabetes, while two others found no difference [40, 41, 42]. We analyzed colon and rectal cancers separately and observed a slightly worse colon cancer survival with diabetes, whereas in rectal cancer, diabetes was associated with a modestly higher survival. In a meta‐analysis by Zhu et al., the association between diabetes and cancer survival was analyzed separately for colon and rectal cancer. They found slightly lower colon cancer survival in patients with diabetes, very similar to our results, though their result was not statistically significant, unlike ours. They also found a nonsignificantly reduced cause‐specific survival for rectal cancer associated with diabetes, while our finding indicated a small but significant improvement in survival. Both estimates were statistically compatible, however, with overlapping confidence intervals.

We found no differences in the survival of patients with pancreatic, bladder, kidney, lung, prostate, or nonmelanoma skin cancer. A previous meta‐analysis did not show a survival difference in pancreatic cancer between patients with and without diabetes for unresectable disease or when all stages were included [43]. As a side note, new‐onset diabetes may occasionally be the first sign of pancreatic cancer, in which case patients are often diagnosed at a more advanced stage and have poorer survival compared with nondiabetic patients [44]. A recent meta‐analysis by Dong et al. reported reduced survival associated with diabetes in bladder cancer patients who underwent radical cystectomy. Their study population was selected based on the treatment modality, which may explain the differences in results. No study was found that assessed all treatment modalities combined. Although the effect of diabetes on kidney cancer survival has been widely studied over the past decade, the results across different meta‐analyzes and original studies remain inconsistent [45]. So far, no clear conclusion has been reached. We found no difference in renal cancer survival between patients with and without diabetes. In lung cancer, our results showed no differences between the groups, which is consistent with the earlier literature [46, 47].

Regarding prostate cancer, previous studies were consistent with our finding that diabetes does not significantly affect prostate cancer survival [48, 49]. However, a Finnish study reported reduced survival among prostate cancer patients with hyperglycemia [50]. Yet, their study question clearly differs from our study, which focused specifically on patients with diagnosed diabetes, regardless of their glycemic status. Survival from nonmelanoma skin cancer was comparable in patients with diabetes than among those without diabetes, and there is little prior research on this topic. For liver cancer, previous studies suggest substantially poorer survival outcomes in diabetes, which is inconsistent with our results [51, 52]. Previous studies focused on overall survival instead of cancer‐specific survival. However, as liver cancer is highly lethal, this alone does not explain the observed differences. Metabolic dysfunction‐associated steatotic liver disease is common in diabetes, and any hepatic abnormalities may be monitored more closely in patients with diabetes. This could lead to early detection and more favorable outcomes. Also, most previous studies have been conducted in high‐risk populations in Southeast Asia with patients diagnosed in the 1980s and 1990s. These patients probably had less intensive monitoring and follow‐up than those in our setting.

Our study has several strengths, such as a large population‐based cohort and comprehensive data on diabetes and cancer from multiple nationwide registers [53, 54]. This approach helps avoid selection and information bias. Our dataset is very large, with over 1000 cancer cases among individuals with diabetes for seven cancer types and more than 700 cases for each of the 13 sites studied. In contrast, previous studies have typically included only a few hundred such cases, and some meta‐analyzes have been conducted using datasets of similar or smaller sizes. We also had complete follow‐up, which enhanced the validity and reliability of our findings. We were able to cover all the most common cancer types, with several hundred cancer deaths for most sites. While cancer mortality among patients with diabetes has been widely studied, data on cause‐specific survival after cancer diagnosis are still limited for several cancer types.

A major limitation of our study was the lack of data on clinical prognostic factors, such as grade, stage, treatment, and comorbidities. However, these could be regarded as mediating factors in our analysis, as they are likely intermediate steps in the causal chain from diabetes to cancer outcomes. The lack of clinical details on diabetes, such as blood glucose levels, HbA1c measurements, and medications, is also a limitation. The register data did not enable an accurate distinction between Type 1 and Type 2 diabetes. However, the majority of older participants are likely to have Type 2 diabetes [55]. In addition, over 90% of the patients with diabetes in our study were exclusively on oral diabetes medication, a treatment specific to Type 2 diabetes. We solely utilized the baseline characteristics of diabetes mellitus and were unable to exclude individuals in the reference group who were later diagnosed with diabetes. Additionally, like in any population‐based study, there were likely undiagnosed subjects with diabetes in the reference group, even at baseline. Furthermore, the racial and ethnic homogeneity of the Finnish population may limit the generalizability of our findings to other populations.

While diabetes affects the survival of several cancer types, the results are not consistent. Although diabetes can materially worsen cancer prognosis, better glycemic control may reduce excess mortality. Moreover, there are indications that some diabetes medications, particularly metformin, may lower cancer mortality in patients with diabetes. Future studies should focus on the biological and metabolic factors influencing cancer survival in patients with diabetes and on how treatment strategies can be optimized to reduce excess cancer mortality in this patient group.

## Author Contributions

**Anssi Auvinen:** conceptualization, investigation, funding acquisition, writing – review and editing, methodology, validation, project administration, supervision, resources. **Petrus Lohi:** conceptualization, investigation, writing – original draft, writing – review and editing, formal analysis, visualization, methodology, funding acquisition. **Jari Haukka:** conceptualization, investigation, methodology, validation, visualization, writing – review and editing, software, formal analysis, supervision, project administration. **Leo Niskanen:** conceptualization, writing – review and editing, validation, project administration, supervision, investigation, data curation.

## Funding

This work was supported by the EU [European Commission, Seventh Framework Program, grant agreement number 282526]. The first author also received personal grants from the Cancer Foundation Finland and the Diabetes Research Foundation, which are gratefully acknowledged.

## Ethics Statement

The study plan was approved by the Faculty of Medicine, University of Helsinki Ethical Committee on January 17, 2012 (Ref 02/2012). All data from the registers were anonymous; the researchers had no information about their identities, and there was no contact with the study population. According to Finnish law, no consent from patients is required for purely register‐based studies.

## Conflicts of Interest

The authors declare no conflicts of interest.

## Supporting information


**Figure S1:** Kaplan–Meier curves for cancer‐specific survival by diabetes status (solid line = no diabetes, dashed line = diabetes), presented separately for each cancer type: (A) stomach, (B) colon, (C) rectum, (D) liver and intrahepatic bile ducts, (E) pancreas, (F) bronchus and lung, (G) hematopoietic and reticuloendothelial systems, (H) skin, (I) breast, (J) corpus uteri, (K) prostate, (L) kidney, (M) bladder, (N) unknown primary site, and (O) other.

## Data Availability

The data that support the findings of this study are available from the corresponding author upon reasonable request.
