# Supplementary figures and images for: Diabetes and Cancer‐Specific Survival: A Nationwide Population‐Based Cohort Study Across Multiple Cancer Sites
Source: Cancer Rep (Hoboken). 2026 Jun 18;9(6):e70615. doi: 10.1002/cnr2.70615 (PMC13278826; doi:10.1002/cnr2.70615)

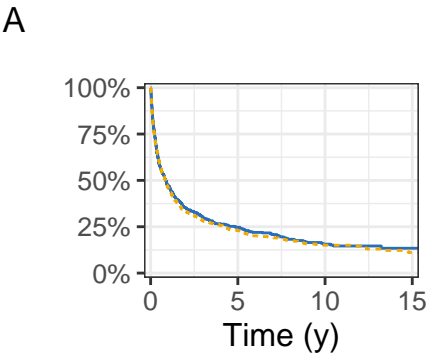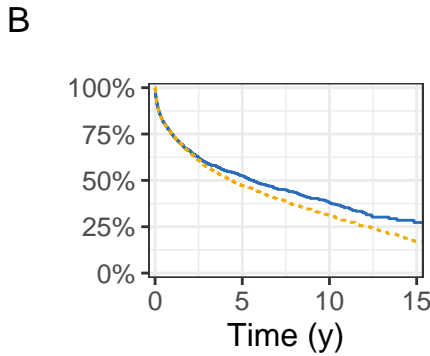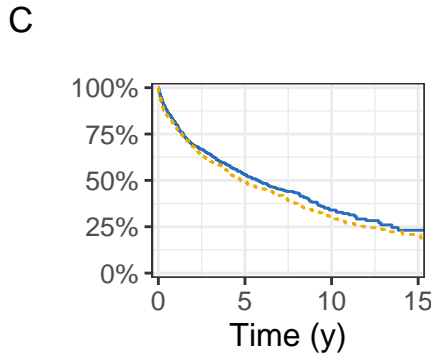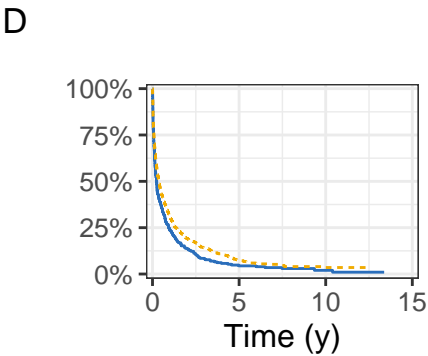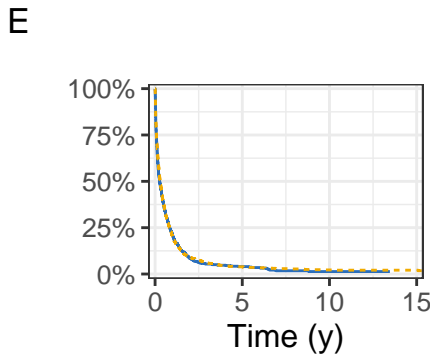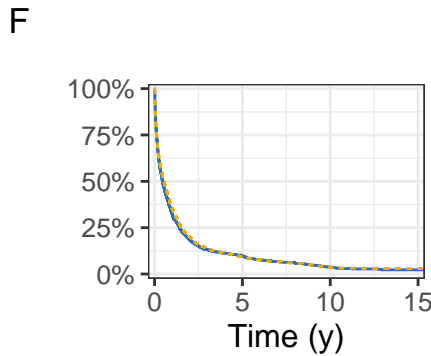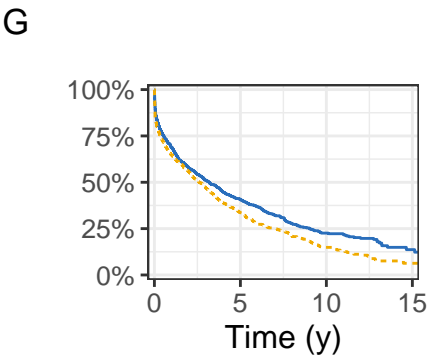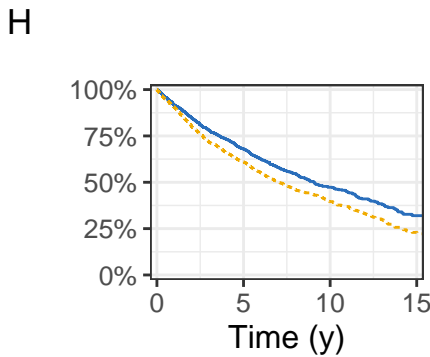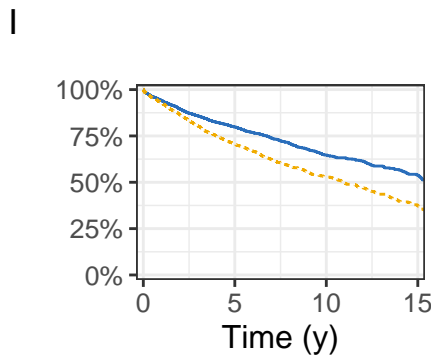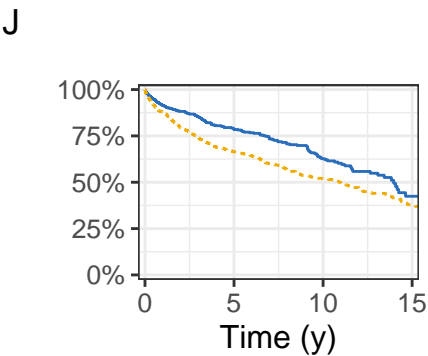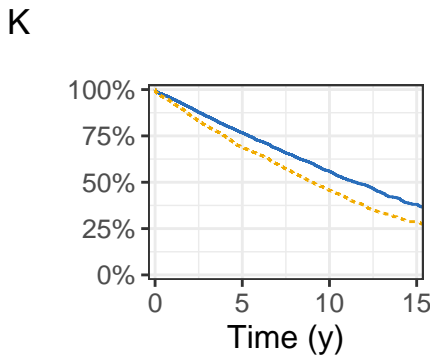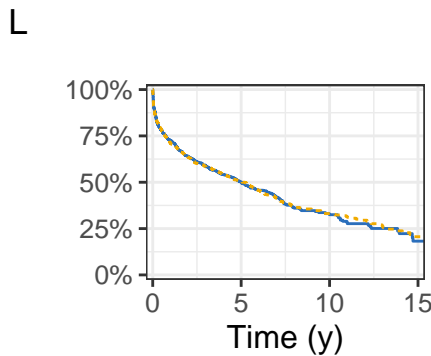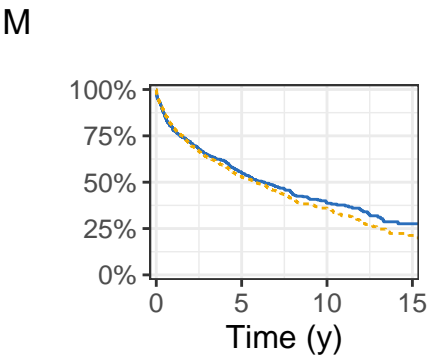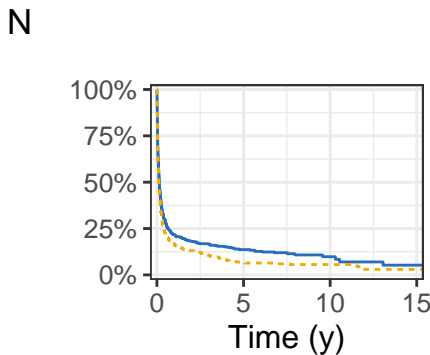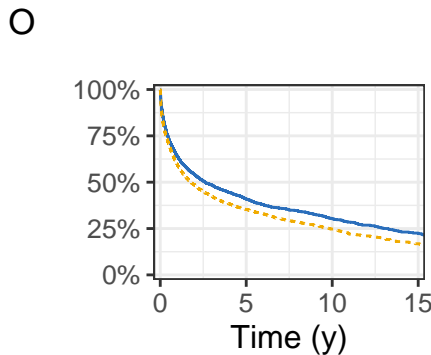

Supplement: Supplementary file 1 — Figure S1: Kaplan–Meier curves for cancer‐specific survival by diabetes status (solid line = no diabetes, dashed line = diabetes), presented separately for each cancer type: (A) stomach, (B) colon, (C) rectum, (D) liver and intrahepatic bile ducts, (E) pancreas, (F) bronchus and lung, (G) hematopoietic and reticuloendothelial systems, (H) skin, (I) breast, (J) corpus uteri, (K) prostate, (L) kidney, (M) bladder, (N) unknown primary site, and (O) other. [file CNR2-9-e70615-s001.pdf]
